# A Child With Acute Hematogenous Osteomyelitis of the Distal Fibula and Infective Endocarditis

**DOI:** 10.7759/cureus.35429

**Published:** 2023-02-24

**Authors:** Jacem Saadana, Oussama Lassioued, Said Abid, Maha Ben Mansour, Abderrazek Abid

**Affiliations:** 1 Orthopaedics and Traumatology, Fattouma Bourguiba University Hospital, Monastir, TUN; 2 Anesthesiology and Reanimation, Fattouma Bourguiba University Hospital, Monastir, TUN

**Keywords:** pediatrics, infective endocarditis, staphylococcus aureus, fibula, acute hematogenous osteomyelitis

## Abstract

Acute hematogenous osteomyelitis (AHO) commonly interests the pediatric population. It typically affects the metaphyses of long tubular bones. However, the fibula is rarely involved. Regarding the hematogenous inoculation, this infection may be associated with distant foci.

Herein, we present the case of a 10-year-old girl who was initially diagnosed with atypical AHO of the distal fibula complicated by a subperiosteal abscess. Upon admission, the child showed a severe septic condition, including embolic infective endocarditis (IE), which was responsible for multiple cerebral, renal, and splenic loci. AHO was successfully resolved with appropriate intravenous antimicrobial treatment targeting Staphylococcus aureus, surgical drainage, and debridement. Due to the complexity of the lesions and the embolic nature, the IE was also managed operatively with a mechanical prosthesis.

The distal fibula is a rare and challenging location for AHO. EI co-infection should always be sought and suspected because, in such instances, it will genuinely complicate diagnostic and therapeutic management.

## Introduction

Osteomyelitis is the inflammation of bone marrow and adjacent tissue, usually caused by pyogenic bacteria. It typically involves the metaphysis of the long bones in pediatric patients via hematogenous dissemination. It is classified as acute if the symptomatology is less than two weeks old at the time of diagnosis [1]. Only a few papers have been published regarding acute hematogenous osteomyelitis (AHO) of the fibula (less than 10%). Furthermore, the clinical presentation is often misleading [2,3]. AHO can be associated with distant extraosseous infection sites, particularly infective endocarditis (IE), which may complicate the situation in terms of diagnosis and treatment [4]. We illustrate the case of a girl treated for AHO at an unusual location (the distal fibula) with an embolic IE to discuss the clinical condition, the correlating physiopathology, and the management.

## Case presentation

A healthy 10-year-old girl presented to our emergency department with a two-day history of nonspecific left ankle pain and high fever peaks of up to 39.5 °C. Her parents did not note any inciting trauma, but two weeks earlier, she had been treated for a presumed viral infection of the upper respiratory tract.

**Figure 1:**
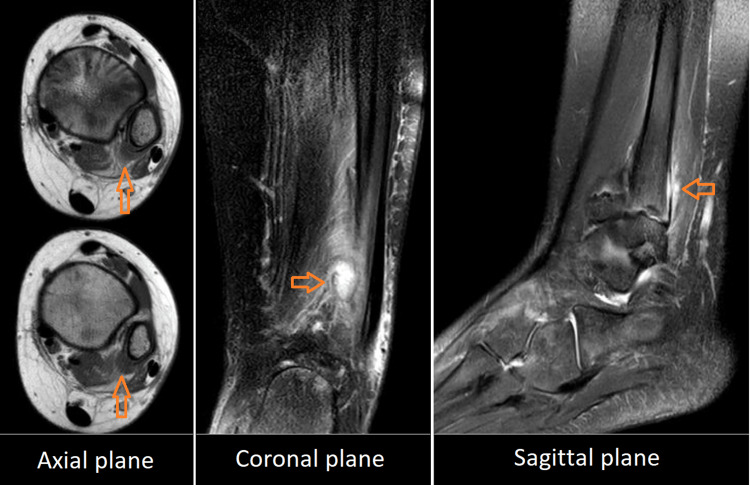
Ankle MRI reveals AHO of the distal fibula complicated by a subperiosteal abscess that has spread to adjacent soft tissue

On physical examination, she had an undistinguished limp and a tenderness point on the posteromedial aspect of the left lateral malleolus metaphysis. Otherwise, no local inflammatory signs were noted, and the ankle range of motion was not restricted or tender. Laboratory analysis tests, including the white blood cell count and the C-reactive protein level, were remarkably elevated. Standard radiographs and ultrasounds of the ankle appeared normal. A further imaging study with magnetic resonance imaging (MRI) was subsequently performed, demonstrating a subperiosteal abscess of the posteromedial aspect of the distal left fibula metaphysis with an extension to adjacent soft tissue, establishing the diagnosis of a locally complicated AHO (Figure 1).

Intravenous antimicrobial therapy with cefazolin and gentamicin was empirically initiated directly after blood cultures were taken. Therefore, surgical treatment was immediately indicated. During the pre-anesthetic examination, mitral systolic murmurs and clicks were found. Echocardiography revealed mitral insufficiency with prolapsed, thickened valve leaflets and hyperechoic vegetations suggestive of IE.

**Figure 2:**
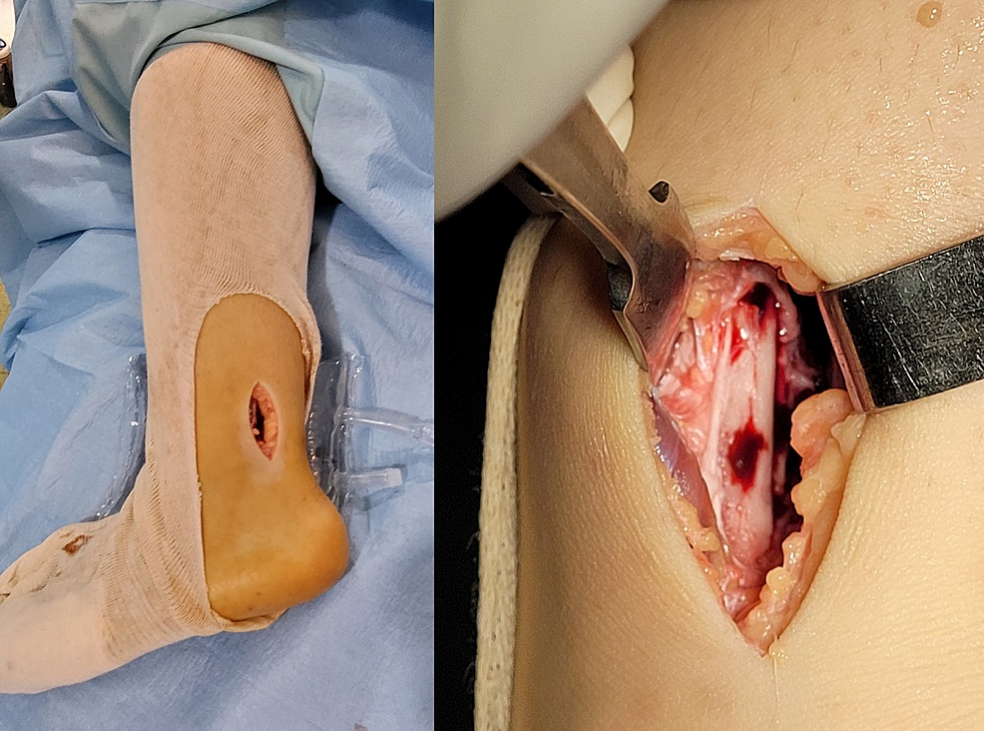
Subperiosteal abscess drainage and lavage via a posterolateral approach

The patient underwent open surgical drainage of the abscess through a posterolateral approach in the lateral position. The procedure began with periosteum excision, bacteriological sampling, cortical drilling, and extensive medullary bone and soft tissue irrigation (Figure 2).

**Figure 3:**
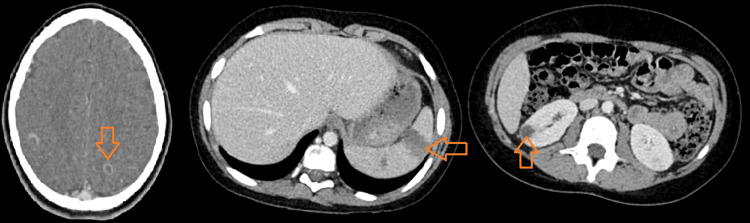
Axial body scan slides showing, respectively, cerebral, splenic, and renal abscesses

Finally, a standard fashion closure was performed, and a negative suction drain was left for 72 hours. Postoperative recovery was uneventful. A body scan revealed multiple septic cerebral, renal, and splenic abscesses that do not require surgical treatment (Figure 3).

These findings suggested the diagnosis of embolic IE of the mitral valve that was responsible for multiple septic localizations, including osteomyelitis. Microbiological examination of blood and sample cultures yielded methicillin-sensitive Staphylococcus aureus. This germ was susceptible to all tested antimicrobials.

On the 10th day, the child was transferred to the pediatric cardiothoracic surgery department. Due to the complexity of the lesions, she underwent mitral valve replacement with a mechanical prosthesis. The antibiotic therapy was changed to intravenous oxacillin, teicoplanin, and imipenem/cilastatin for three weeks, followed by cefazolin for three additional weeks.

Postoperatively, the child showed clinical and laboratory improvement. The radiological evaluation showed no sign of secondary bone lesions or pandiaphysitis. She resumed a normal gait with no restrictions and was discharged eight weeks after the initial surgery.

At the six-month follow-up, no recurrence of the symptoms was observed, the left ankle had a full range of motion, and the cardiovascular exam was without abnormalities. In addition, blood test results were within normal limits, and X-rays showed ad-integrum lateral malleolar bone healing without any sign of local complications.

## Discussion

Acute infective osteomyelitis in children is usually caused by microorganisms introduced to the bone through hematogenous delivery secondary to symptomatic or asymptomatic septicemia in otherwise healthy individuals [2,5]. In our patient, we believe that an oropharyngeal infection is the source of bacteremia.

A history of trauma might be considered a predisposing factor for AHO, as trauma is commonly thought to contribute to metaphyseal capillary network bleeding. Therefore, it facilitates bacteria breeding and infection spreading into the bone. The trauma report was prevalent in nearly 30% of children with osteomyelitis [2,6,7].

The causative germ varies depending on the child's age. Staphylococcus aureus is currently the most frequent pathogen, similar to our patient, and accounts for almost 70-80% of the case series, followed by Streptococcus pneumoniae (whose incidence has decreased significantly since the introduction of the conjugate vaccine) and Streptococcus pyogenes. Except in the neonatal period, Gram-negative rods are much less common [7-10].

AHO primarily affects children's long tubular bones in the metaphyseal region due to the increased blood supply and slow flow through the undeveloped collateral circulation [9]. Compared to the upper extremity, the lower extremity is somewhat more involved. The AHO concerns the femur at 25%, the tibia at 24%, and the humerus at 13% [1,11]. The fibula is rarely affected in the pediatric population (4-10%) [3]. The distal fibula is concerned more often than the proximal metaphysis [2,6,12].

AHO in the distal fibula is frequently associated with a misleading clinical presentation and requires careful diagnostic measures and assessments [2]. Typically, we find pain and sparing of the affected limb during the clinical examination. The localization of pain can be difficult when lameness or functional impotence is the only clinical sign. Local inflammatory symptoms, such as swelling, redness, warmth, and joint effusion, are observed in about 70% of cases and more frequently in arthritis than in osteomyelitis. Fever is found in only 60% of AHO cases [3,8,12,13]. In this study, we had an AHO with an unusual site and minor symptoms that could lead to overlooking the diagnosis.

Several imaging modalities have been used to facilitate the diagnosis of AHO and to exclude other diseases, such as fractures and tumors, including plain radiographs, ultrasound, skeletal scintigraphy, computed tomography (CT), and MRI, which has a significantly higher sensitivity (97-100%) and specificity (92%). In addition, MRI is the most appropriate method for the early detection of bone marrow changes and for evaluating local complications (abscesses, joint effusions, and soft tissue extensions) [1,7,11,13,14].

A generalized clinical examination is essential to investigate distant concomitant infections, whether osteoarticular or in other systems (respiratory, cardiovascular, and neurological). In addition, the rare localization of AHO should draw attention to the need to look for severe sepsis and screen for a possible immunocompromised status [11].

In our patient, thanks to the pre-anesthetic consultation indicated for classic surgical drainage of the subperiosteal abscess, we considered the mitral murmur evocative of IE, which was confirmed directly by echocardiography. Later, the CT scan revealed the presence of extra-cardiac septic locations (cerebral, renal, and splenic).

Children are rarely diagnosed with IE compared to adults. In recent studies, the incidence of pediatric IE has been estimated to be approximately 0.43-0.69 cases per 100,000 children per year. It usually occurs as a complication of congenital and rheumatic heart diseases. However, it is a significant cause of morbidity and mortality in children [4,15,16].

Musculoskeletal symptoms, including myalgia and osteoarticular pain, are common findings in IE and can be the child’s primary complaint. Therefore, this makes it difficult for the clinician to select those patients who need investigation for osteomyelitis and to avoid misdiagnosis [4,17].

The correlation between AHO and IE is polymorphic due to heterogeneous pathophysiology: either these localizations are synchronic following bacteremia, or frequently, osteomyelitis is secondary to IE as a distant infection. Extracardiac endocarditis complications are most notably the result of embolic phenomena from vegetation. It may be present in 10-40% of children. Depending on embolic migration, they may involve the cerebral, pulmonary, renal, splenic, and osteoarticular loci. Osteomyelitis is thought to occur as a complication in 6% of endocarditis cases [4,15,16].

In children, standard AHO treatment entails immediate empiric antibiotic therapy [5]. Antibiotics should aim principally at Staphylococcus aureus, the most common microorganism, and must be initiated immediately after collecting specimens. Appropriate antibiotic treatment has proven efficacious in 90% of AHO cases [1,13].

Surgical management should be considered whenever there is no response to intravenous antibiotic therapy, symptom persistence, a subperiosteal abscess, or another soft tissue abscess. The procedure consists of drainage and the debridement of all affected tissues [1,11].

The absence of response to empiric antibiotic therapy may suggest the implication of an uncommon microbial agent that is not susceptible to S. aureus antimicrobials. In that case, surgical intervention is indicated to confirm the diagnosis, determine the causative germ, and enhance the antimicrobial therapy [2].

The recommended antibiotic regimes for AHO range from four to six weeks, prolonged depending on the causative organism, type of surgical evacuation, site of infection, associated complications, and clinical and laboratory response [13].

Treatment of AHO associated with IE in the pediatric population should be multidisciplinary and provided with the cooperation of infectiologists, cardiologists, and cardiac surgeons [15,17]. Before germ identification, empiric vancomycin and gentamicin therapy are recommended after blood cultures are obtained to provide complete coverage against the most common pathogens of EI, S. aureus, and viridans streptococci. The antibiotic regimen will be tailored later based on specific pathogen susceptibility profiles [15].

Prolonged intravenous bactericidal antibiotics may be mandatory to eradicate offending agents and prevent further endocardial complications, especially in patients with prosthetic valves. Clinical and biological responses should be carefully monitored to determine whether antibiotics or surgical intervention are required [4,15].

Surgery should be well thought out in patients with a high risk of embolic events, prosthetic valve endocarditis, intractable heart failure, and uncontrolled infection. Based on the extent of IE damage to the valve, a surgical intervention (repair or replacement) will be determined. Management decisions for EI are mainly guided by the American Heart Association (AHA), which has published comprehensive guidelines for antimicrobial and surgical treatment [4,16].

## Conclusions

AHO of the distal fibula is a rare clinical condition in pediatric patients. A careful initial examination is mandatory to avoid misdiagnosis and detect concomitant distant infections. The diagnosis of AHO should serve as a warning finding for the physician to suspect EI coexistence and vice versa. This association is severe and can lead to potentially devastating morbidity and mortality. Thus, it requires prompt and multidisciplinary management involving pediatricians, infectiologists, orthopedic surgeons, radiologists, cardiologists, and cardiac surgeons.
